# Transcriptomic Responses and Putative Mechanisms of Nicotinamide-Mediated Saline–Alkaline Tolerance in Soybean

**DOI:** 10.3390/metabo16090649

**Published:** 2026-09-04

**Authors:** Qi Zhang, Fanyu Meng, Haoxuan Sun, Yajie Yin, Guoling Ren

**Affiliations:** 1 School of Biological Engineering, Daqing Normal University, No. 23, Xijia Road, Ranghulu District, Daqing 163712, China; dqnuzhangqi@126.com (Q.Z.); 13134507780@163.com (F.M.); sunhaoxuan167@163.com (H.S.); 2 College of Bioengineering, Daqing Normal University, Daqing 163712, China; 3 Heilongjiang Provincial Key Laboratory of Oilfield Applied Chemistry and Technology, Daqing 163712, China

**Keywords:** nicotinamide, soybean, saline–alkali stress, transcriptome, *PM1*

## Abstract

**Highlights:**

**What are the main findings?**

- Exogenous nicotinamide at 50 mg/L most effectively enhances soybean saline–alkaline tolerance, significantly improving growth and physiological parameters.Nicotinamide treatment reduces reactive oxygen species (ROS) accumulation and restores Na^+^/K^+^ homeostasis under saline–alkaline stress.
- Transcriptomic analysis reveals that differentially expressed genes under non-optimal treatments are enriched in protein folding, cytochrome P450, and endoplasmic reticulum (ER) processing pathways.

**What are the implications of the main findings?**

- These findings identify optimal nicotinamide concentration for improving crop stress tolerance, providing a practical reference for agricultural application.
- The elucidation of enriched pathways offers mechanistic insights into nicotinamide-mediated stress responses, informing future molecular breeding or genetic engineering strategies for saline–alkaline tolerance in soybean.

**Abstract:**

**Objective:** Saline–alkali stress (composed of neutral and alkaline salts) severely constrains soybean productivity. Although nicotinamide can enhance plant salt tolerance, its most effective concentration and molecular basis in soybean remain unclear. This study aimed to systematically investigate the regulatory effect of exogenous nicotinamide on soybean saline–alkaline tolerance and to identify its candidate genes and putative mechanisms under salt-stressed conditions. **Methods:** Soybean cultivar ‘Hefeng 25’ seedlings were exposed to 200 mmol/L mixed salt stress and treated with nicotinamide at 0 (CK), 10, 50, and 200 mg/L. Growth parameters, ROS levels, ion contents, photosynthetic pigments, osmoregulatory substances, and antioxidant enzyme activities were measured. Transcriptome sequencing and RT-qPCR were performed to identify differentially expressed genes (DEGs), followed by enrichment analysis. **Results:** Exogenous nicotinamide significantly improved soybean saline–alkaline tolerance relative to the salt-stressed CK group, with 50 mg/L (SA2) showing the most pronounced physiological improvements among all tested concentrations. SA2 treatment enhanced plant height, biomass, K+ content, and antioxidant enzyme activities while reducing Na+ accumulation and ROS levels compared with CK. Transcriptome analysis identified *PM1* as the shared gene across the five non-most effective comparisons. Enrichment analysis implicated DEGs primarily in ko03110 (chaperones and folding catalysts), ko00199 (cytochrome P450), and ko04141 (protein processing in the endoplasmic reticulum). RT-qPCR confirmed that *PM1*, P450 genes (*CYP74A1*, *CYP83D1*), HSPs (*HSP23*, *HSP18*), and ROS scavengers (*NAC1*, *GSTU43*) were upregulated under non-most effective doses (SA1/SA3) but downregulated to levels comparable to the stressed CK in SA2; conversely, SA2 specifically restored the expression levels of stress-repressed *WRKY57* and *LBD15*. Transcriptomically, SA2 exhibited a profile most similar to the saline–alkali-stressed CK group, with the fewest number of differentially expressed genes among all treatments, whereas non-optimal nicotinamide concentrations were associated with extensive transcriptional changes in ER protein processing, detoxification-related, and oxidative stress-related pathways. The limited transcriptional changes in SA2 indicate greater transcriptional similarity to the stressed control, which paralleled its superior physiological performance. **Conclusions:** Nicotinamide effectively alleviates soybean saline–alkaline stress at 50 mg/L, with the most significant physiological benefits and the fewest transcriptional changes relative to the stressed control. The non-most effective nicotinamide concentrations show extensive stress-related transcriptional reprogramming in protein folding, detoxification, and stress-response pathways relative to the salt-stressed control, putatively implicating ER stress, detoxification, and oxidative stress-related responses at the transcript level, with *PM1* acting as a potential candidate transcriptional marker of dose-dependent transcriptional regulation under saline–alkali stress.

## 1. Introduction

Soil salinization is a major environmental constraint limiting plant growth, development, and productivity [1,2]. Unlike neutral salt stress (predominantly NaCl), which mainly causes ionic and osmotic damage, saline–alkali stress is a composite stress condition characterized by the coexistence of neutral salts (e.g., NaCl, Na_2_SO_4_) and alkaline salts (e.g., NaHCO_3_, Na_2_CO_3_), which not only induces ion toxicity and osmotic imbalance but also elevates environmental pH, causing additional alkaline damage and disrupting intracellular pH homeostasis. Approximately 7% of the world’s land area is affected by varying degrees of salinization [3]. Under saline–alkali stress, ion toxicity, osmotic stress, and oxidative damage collectively disrupt plant cellular structure, inhibit photosynthesis, and impair primary metabolite synthesis, ultimately resulting in yield reduction or even plant death [4]. Among all developmental stages, the germination and seedling phases are particularly vulnerable to saline–alkali stress, which can cause irreversible damage [5]. As an economically important crop in China, soybean (*Glycine max* L.) is also severely affected by saline–alkali stress [6,7,8]. Soybean (*Glycine max*) is highly sensitive to saline–alkali stress due to three interconnected deficiencies: inefficient root Na^+^ exclusion and xylem sequestration, extreme vulnerability to iron deficiency under alkaline pH, and the exceptional fragility of its symbiotic nitrogen-fixation system. These constraints collectively impair ion homeostasis, photosynthetic efficiency, and nodule function, rendering soybean far less tolerant than most other crops. Such stress induces excessive accumulation of reactive oxygen species (ROS) in soybean cells, which compromises plasma membrane integrity, disrupts protein function, and jeopardizes genome stability, thereby inhibiting both growth and biomass accumulation [9]. Consequently, enhancing saline–alkaline tolerance is of great significance for the development of sustainable soybean farming practices.

Nicotinamide, the bioactive form of vitamin B3, is a key precursor for the biosynthesis of nicotinamide adenine dinucleotide (NAD^+^) and nicotinamide adenine dinucleotide phosphate (NADPH) in plants, and is extensively involved in fundamental physiological processes, including energy metabolism, redox homeostasis, and maintenance of cellular redox balance [10,11,12]. As an essential substrate for poly(ADP-ribose) polymerase (PARP) and sirtuin (SIRT) deacetylases, nicotinamide actively participates in the regulation of NAD^+^ consumption and turnover, thereby influencing ATP production, mitochondrial respiration, and the activity of NAD^+^-dependent signaling enzymes that couple metabolic status with stress-responsive transcriptional programs [12,13]. Beyond its coenzyme precursor function, nicotinamide exhibits intrinsic bioactivities, including direct scavenging of reactive oxygen species (ROS), chelation of transition metal ions to suppress Fenton-type chemistry, and modulation of calcium-dependent signal transduction pathways, collectively contributing to its protective effects against oxidative damage [13]. Accumulating evidence indicates that exogenous application or promotion of endogenous synthesis of nicotinamide can significantly enhance plant saline–alkaline tolerance through the coordinated upregulation of antioxidant enzyme systems and osmoprotectant biosynthesis [13,14]. For example, Santos et al. reported that the combined application of nicotinamide, proline, and salicylic acid exerted synergistic effects, markedly enhancing carbon assimilation efficiency, preserving photosynthetic pigment stability, and ultimately improving plant salt stress tolerance [14]. Despite these promising findings, the optimal concentration and regulatory mechanisms of nicotinamide in soybean under saline–alkaline stress remain largely unexplored. Most previous studies have focused on physiological responses, while the transcriptional reprogramming underlying nicotinamide-mediated stress alleviation has not been systematically characterized. Moreover, it remains unclear whether the protective effects observed in other species or under neutral salt stress are directly transferable to soybean under mixed saline–alkaline conditions. Elucidating the mechanisms by which nicotinamide alleviates saline–alkali stress in soybean is, therefore, of considerable value for improving soybean productivity and ensuring food security.

RNA sequencing (RNA-seq) has emerged as a transformative tool for plant stress biology, offering several distinct advantages over conventional gene-by-gene approaches. Its high-throughput nature allows genome-wide monitoring of transcript abundance with exceptional sensitivity and quantitative accuracy, capturing both robust and subtle expression changes across the entire transcriptome. As an unbiased screening platform, RNA-seq enables the discovery of novel transcripts, alternative splicing events, and previously uncharacterized stress-responsive genes without requiring prior sequence information or candidate gene selection. Moreover, the resulting expression matrices can be subjected to functional enrichment analyses, co-expression network construction, and pathway mapping, thereby revealing the hierarchical regulatory architecture and the interconnected signaling cascades that underlie complex physiological traits such as stress tolerance. However, several key knowledge gaps remain regarding nicotinamide application in soybean under saline–alkaline stress. First, the optimal dosage of nicotinamide for soybean under mixed saline–alkaline conditions has not been systematically evaluated. Second, whether the protective effects observed in other species or under neutral salt stress are transferable to soybean—a crop with inherently high sensitivity to alkaline pH and ion toxicity—remains unclear. Third, the global transcriptional responses underlying nicotinamide-mediated stress alleviation in soybean have not been characterized. Therefore, this study was designed to address these gaps by: (i) identifying the most effective nicotinamide concentration through comprehensive physiological assessment; (ii) characterizing the soybean-specific transcriptional landscape under mixed saline–alkaline stress; and (iii) exploring the putative molecular pathways involved in nicotinamide-mediated stress alleviation. In this study, the soybean cultivar ‘Hefeng 25’ was used as the experimental material to evaluate the effect of nicotinamide on saline–alkaline tolerance in soybean. Transcriptomic analysis was further employed to identify potential key targets and to explore the underlying molecular mechanisms. These findings contribute to a better understanding of nicotinamide-mediated stress alleviation and may inform future strategies for improving crop performance under saline–alkaline conditions. The soybean cultivar ‘Hefeng 25’ was selected as the experimental material for the following reasons. This cultivar is widely cultivated in the saline–alkali-affected regions of Heilongjiang Province, China, and exhibits moderate sensitivity to saline–alkaline stress, making it an appropriate representative for evaluating ameliorative strategies under local soil conditions. Moreover, its relatively stable agronomic traits and well-characterized physiological responses to environmental stress facilitate reproducible experimental assessments. The use of ‘Hefeng 25’, therefore, provides both practical relevance for regional soybean production and a reliable system for investigating the regulatory effects of nicotinamide on saline–alkaline tolerance.

## 2. Methods

### 2.1. Plant Materials and Treatment

Seeds of the soybean cultivar ‘Hefeng 25’ were sown in pots containing a soil mixture of cultivated soil, humus soil, and sand at a ratio of 2:1:1 (*v*/*v*). Seedlings were grown in a greenhouse under controlled conditions at 25 ± 1 °C with a 16/8 h light/dark photoperiod. In the V1 stage (unifoliolate leaf fully expanded), saline–alkali stress was imposed by irrigating seedlings with 100 mL of a mixed salt solution (NaCl, NaHCO_3_, Na_2_CO_3_, and Na_2_SO_4_ at a molar ratio of 1:1:9:9) at a final concentration of 200 mmol/L. The pH and electrical conductivity (EC) of this salt mixture have been previously characterized as approximately 8.6 and 16.8 dS/m, respectively, under equivalent concentrations [6]. Each pot received 100 mL per application, applied once every two days for a total of three applications (cumulative 300 mL per pot). The control group (CK) received an equal volume of tap water following an identical irrigation schedule. Stress establishment was confirmed on Day 7 after the first salt irrigation, as indicated by visible leaf wilting and reduced turgor, consistent with previous reports using the same salt treatment [6]. To establish an appropriate dosage range for nicotinamide application in soybean, we conducted preliminary experiments with a broader concentration gradient (0, 5, 10, 25, 50, 100, 200, and 500 mg/L) under saline–alkaline stress. Based on these trials, 10 mg/L was identified as a sub-effective concentration with marginal physiological improvements; 50 mg/L produced the most pronounced enhancement in growth and stress tolerance parameters; and 200 mg/L exhibited diminished or adverse effects, indicating a supra-effective response. Therefore, these three concentrations—representing sub-effective, most effective, and supra-effective doses—were selected for further analysis. Nicotinamide solutions at three concentrations—10 mg/L (SA1), 50 mg/L (SA2), and 200 mg/L (SA3)—were applied as a single foliar spray at a volume of 20 mL per pot (approximately 5–7 mL per seedling), ensuring uniform coverage of both adaxial and abaxial leaf surfaces using a hand-held sprayer with a droplet size fine enough to avoid runoff. The spraying was performed on Day 7 after the first salt irrigation (i.e., two days after the final salt application) in the early morning (between 6:00 and 7:00 AM) to minimize evaporation and photodegradation. No surfactant or wetting agent was added to the solutions. The control group (CK) received an equal volume of deionized water sprayed under identical conditions. Each treatment group consisted of 15 pots, with three seedlings per pot, arranged in a completely randomized design (CRD) within the greenhouse. Pots were repositioned randomly every two days to minimize positional effects. All treatments were carried out with three independent biological replicates. Plant samples were collected three days after the final nicotinamide application.

### 2.2. Measurement of Antioxidant Enzymes

Frozen leaf samples (0.5 g) were ground to a fine powder in a pre-chilled mortar with 5 mL of 50 mM phosphate buffer (pH 7.8) containing 1% (*w*/*v*) polyvinylpyrrolidone (PVP) and 1 mM EDTA. The homogenate was centrifuged at 12,000× *g* for 20 min at 4 °C, and the supernatant was collected as the crude enzyme extract for subsequent assays. All enzyme activities were determined spectrophotometrically using three biological replicates. Peroxidase (POD) activity was measured by monitoring the oxidation of guaiacol at 470 nm [15]. Superoxide dismutase (SOD) activity was assayed by its ability to inhibit the photochemical reduction of nitroblue tetrazolium (NBT), with absorbance recorded at 560 nm [16]. Catalase (CAT) activity was determined by monitoring the decrease in absorbance at 240 nm resulting from H_2_O_2_ consumption [17]. Ascorbate peroxidase (APX) activity was measured by tracking the decrease in ascorbate absorbance at 290 nm, following the method described by Wang et al. [18].

### 2.3. Determination of Ion Content

Approximately 0.1 g of sample was pre-treated with water, digested overnight with 98% sulfuric acid, and heated at 280 °C for 1 h with three aliquots of H_2_O_2_ added sequentially until the solution clarified. The digest was then cooled, diluted to 50 mL with deionized water, and analyzed for potassium (K^+^) and sodium (Na^+^) concentrations using a flame photometer (FP640, Shanghai INESA Analytical Instrument Co., Ltd., Shanghai, China) [19]. Hydrogen peroxide (H_2_O_2_) content was determined by the potassium iodide spectrophotometric method, with absorbance measured at 390 nm [20]. Superoxide anion (O^2−^·) content was quantified by the hydroxylamine oxidation method, with absorbance recorded at 530 nm [6]. All measurements were performed in triplicate with three independent biological replicates.

### 2.4. Detection of Photosynthetic Pigments

For photosynthetic pigment extraction, leaf samples were cut into small fragments, and approximately 0.1 g of fragments was transferred into 10 mL centrifuge tubes. Each sample was then extracted with 10 mL of absolute ethanol in the dark for 24 h until the tissue was completely decolorized. Pigment concentrations were calculated based on absorbance measurements at 665, 649, and 470 nm using a UV-Vis spectrophotometer [21]. All measurements were performed in triplicate with three independent biological replicates.

### 2.5. Determination of Osmoregulation Products

Soluble protein content was determined by the Coomassie Brilliant Blue G-250 method [8]. Briefly, 1 mL of enzyme extract was mixed thoroughly with 5 mL of Coomassie Brilliant Blue G-250 solution, and the absorbance was measured at 595 nm after 2 min. A standard curve was constructed using bovine serum albumin (BSA) as the standard, with concentrations ranging from 0 to 100 µg/mL (0, 10, 20, 40, 60, 80, and 100 µg/mL). The soluble protein content was calculated from the standard curve and is expressed as mg/g fresh weight (FW). Soluble sugar content was quantified by the anthrone method [18]. Approximately 0.5 g of leaf sample was weighed into test tubes, followed by the addition of 15 mL of distilled water. The mixture was incubated for 20 min, after which 1 mL of the extract was mixed with 5 mL of anthrone reagent and heated for 10 min. Absorbance was then measured at 620 nm. A standard curve was prepared using glucose as the standard, with concentrations ranging from 0 to 100 µg/mL. The soluble sugar content was calculated from the standard curve and is expressed as mg/g FW. Proline content was determined by the acid ninhydrin colorimetric method [22]. Fresh leaf tissue (approximately 0.5 g) was cut into pieces and homogenized with 5 mL of 3% sulfosalicylic acid. The homogenate was boiled in a water bath, and the supernatant was collected after centrifugation at 10,000× *g* for 15 min at 4 °C. The supernatant was then mixed with 2.5% acid ninhydrin reagent and glacial acetic acid at a volume ratio of 1:1:1. After heating and cooling, 4 mL of toluene was added, and the mixture was then thoroughly shaken to facilitate extraction. Following phase separation, the upper toluene phase was collected, and its absorbance was measured at 520 nm. A standard curve was constructed using proline as the standard, and the proline content is expressed as µg/g FW. All determinations were performed in triplicate with three independent biological replicates.

### 2.6. Evaluation of Nicotinamide Nucleotides

The concentrations of NAD^+^, NADH, NADP^+^, and NADPH in leaf samples were evaluated by enzymatic coupling to specific coenzyme diaphorases with DCPIP reduction, utilizing modifications of Yamamoto’s procedure [23]. Briefly, fresh leaf samples (0.5 g) were rapidly frozen in liquid nitrogen and ground to a fine powder in a pre-chilled mortar. Nucleotides were extracted by homogenizing the powder with 5 mL of 0.1 M HCl (for NAD^+^ and NADP^+^) or 0.1 M NaOH (for NADH and NADPH) to selectively stabilize the oxidized and reduced forms, respectively. The homogenates were heated at 60 °C for 5 min, then neutralized with 0.1 M HCl or NaOH, and centrifuged at 12,000× *g* for 10 min at 4 °C. The resulting supernatants were used directly for the enzymatic assays. For quantification, oxidized forms (NAD^+^ and NADP^+^) and reduced forms (NADH and NADPH) were measured separately using specific cycling reactions. The reaction mixture contained 50 mM Tris-HCl buffer (pH 8.0), 0.2 mM DCPIP, 1 mM phenazine methosulfate (PMS), 1 mM EDTA, and 0.5 mM of the respective substrate (e.g., ethanol for NAD^+^, glucose-6-phosphate for NADP^+^). Enzymatic cycling was initiated by adding the corresponding dehydrogenases (alcohol dehydrogenase for NAD^+^; glucose-6-phosphate dehydrogenase for NADP^+^), and the rate of DCPIP reduction was monitored spectrophotometrically by measuring the decrease in absorbance at 600 nm over 10 min. All assays were performed at 25 °C in a final volume of 1 mL, and the reaction rates were compared with standard curves prepared using known concentrations of authentic NAD^+^, NADH, NADP^+^, and NADPH (0–200 µM). Nucleotide contents were calculated from the linear regression equations of the standard curves and are expressed as nmol per gram fresh weight (nmol/g FW). The ratios of NAD^+^/NADH and NADP^+^/NADPH were then calculated from the respective concentrations. All determinations were performed with three independent biological replicates, and each sample was assayed in triplicate.

### 2.7. Transcriptomics Analysis

#### RNA Extraction and Sequencing

For each biological replicate, leaf tissues were collected from three randomly selected seedlings per treatment and pooled for RNA extraction [24]. Total RNA was extracted from leaf samples of CK, SA1, SA2, and SA3 seedlings using TRIzol reagent (Invitrogen, Carlsbad, CA, USA) according to the manufacturer’s instructions. To eliminate genomic DNA contamination, all RNA samples were treated with DNase I (RNase-free, Takara Bio Inc., Kusatsu, Shiga, Japan) following the manufacturer’s instructions. RNA purity, integrity, and concentration were assessed using a NanoDrop 2000 spectrophotometer (Thermo Scientific, Waltham, MA, USA) and an Agilent 2100 Bioanalyzer (Agilent Technologies, Santa Clara, CA, USA). Library construction was performed using the NEBNext Ultra RNA Library Prep Kit (New England Biolabs, Ipswich, MA, USA) following the manufacturer’s protocol. Transcriptome sequencing was performed on the Illumina NovaSeq 6000 platform (Illumina, San Diego, CA, USA). Raw sequencing data were quality-filtered using FASTP software (v0.20.0) with default parameters (removing adapter sequences, filtering out reads with ambiguous bases > 5%, and discarding reads with a quality score < Q15). Clean reads were then aligned to the soybean reference genome using HISAT2 (v2.2.1) with default parameters. The expression levels of protein-coding genes were calculated as FPKM (Fragments Per Kilobase of transcript per Million mapped reads) values. The raw sequencing data have been deposited in the Science Data Bank under the DOI: 10.57760/sciencedb.42114 (private access link for reviewers: https://www.scidb.cn/s/6rUzM3 (accessed on 5 July 2026).

To assess transcriptome quality and reproducibility, the following quality metrics were monitored: (i) RNA quality was ensured by selecting only samples with an A260/A280 ratio between 1.8 and 2.1, an A260/A230 ratio ≥ 2.0, and an RNA integrity number (RIN) ≥ 8.0; (ii) poly(A)-enriched mRNA libraries were constructed with a paired-end 150 bp (PE150) read strategy; (iii) the sequencing depth was approximately 6.5 Gb of clean data per sample (~22 million paired-end reads); (iv) clean reads were aligned to the soybean reference genome (*Glycine max* Wm82.a2.v1; GCF_000004515.6), with gene annotations obtained from the corresponding GFF file (Ensembl Plants release 49); (v) the average mapping rate was above 92% across all samples, with uniquely mapped reads confirmed by HISAT2 output; and (vi) all biological replicates showed high consistency, as validated by PCA and pairwise correlation analyses.

### 2.8. Differential Expression Analysis

The “DESeq2” software (v1.38.3) was used to identify DEGs among the SA1 vs. CK, SA2 vs. CK, SA3 vs. CK, SA2 vs. SA1, SA3 vs. SA1, and SA3 vs. SA2 groups with a cutoff value of *p*.adj < 0.05 and |log_2_FC| > 1, respectively, followed by the screening of overlapping genes.

### 2.9. Enrichment Analysis

Based on the DEGs screened among the SA1 vs. CK, SA2 vs. CK, SA3 vs. CK, SA2 vs. SA1, SA3 vs. SA1, and SA3 vs. SA2 groups, Gene Ontology (GO) and Kyoto Encyclopedia of Genes and Genomes (KEGG) pathway enrichment analyses were conducted using the clusterProfiler package (v4.6.0) in R (v4.2.0). GO enrichment analysis was performed to annotate DEGs into three functional categories: biological process (BP), cellular component (CC), and molecular function (MF), using the enrichGO function with the soybean annotation database org.Glycine.max.eg.db and all annotated soybean genes as the background gene set. KEGG pathway enrichment analysis was carried out using the enrichKEGG function, with the soybean genome set as the reference background (species code gmx). Significantly enriched terms and pathways were defined as those with an adjusted *p*-value (*p*.adj) < 0.05. Terms with *p*.adj ≥ 0.05 were not described as enriched.

### 2.10. RT-qPCR Assay

Soybean leaf samples (0.1 g) were collected at the same time points as those used for transcriptome sequencing. Total RNA was extracted using the DP-452 kit (Tiangen Biochemical Technology, Beijing, China) and reverse-transcribed to obtain cDNA, and fluorescent quantitative PCR primers were designed (Table 1). *GAPDH* was used as the internal reference gene for normalization. The reaction was performed using the FP-205 kit (Tiangen Biochemical Technology, Beijing, China) on an ABI 7500 real-time PCR system (Applied Biosystems, Thermo Fisher Scientific, Waltham, MA, USA). Relative gene expression levels were calculated using the 2^−ΔΔCt^ method.

Primer specificity was confirmed by melt-curve analysis and agarose gel electrophoresis, with each primer pair generating a single amplicon of the expected size (Table 1). Amplification efficiencies were evaluated using standard curves generated from a five-fold serial dilution series of pooled cDNA (undiluted, 5-, 25-, 125-, and 625-fold dilutions); the efficiency (E) of each primer pair was calculated as E = 10^(−1/slope) − 1, where the slope was derived from the linear regression of Ct values versus log10 of the relative dilution factor. All primer pairs exhibited amplification efficiencies within the range of 95.8–102.5% with *R*^2^ ≥ 0.99 (Table 1), supporting the applicability of the 2^−ΔΔCt^ method.

### 2.11. Statistical Analysis

All phenotypic, physiological and RT-qPCR data were analyzed using GraphPad Prism 9.0 software (GraphPad Software, Inc., San Diego, CA, USA). All experiments were performed with three independent biological replicates, and each biological replicate consisted of measurements from three technical replicates. Continuous variables are presented as the mean ± standard deviation (SD). For multiple-group comparisons, one-way analysis of variance (ANOVA) was performed, followed by Tukey’s post hoc test for pairwise comparisons. For comparisons between two groups, Student’s *t*-test was applied. A *p*-value < 0.05 was considered statistically significant.

For RNA-seq transcriptome differential expression analysis, raw read count matrices were imported into DESeq2 (v1.38.3) for statistical testing of differentially expressed genes (DEGs). FPKM- and TPM-normalized expression values were only used for visualizations including PCA plots, correlation heatmaps, gene expression boxplots and density curves, and were not adopted for DEG statistical screening. A total of 43,727 genes were detected across all samples. Genes satisfying |log_2_FC| > 1 and adjusted *p*-value (*p*.adj) < 0.05 were defined as significantly differentially expressed (Supplementary Table S2). Sample reproducibility was comprehensively validated via PCA, pairwise Pearson correlation heatmaps, global gene expression distribution plots and genome mapping statistics; all biological replicates showed high consistency and no outlier samples were identified.

## 3. Results

### 3.1. Nicotinamide Treatment Improves the Salt–Alkaline Tolerance of Soybeans

**Figure 1:**
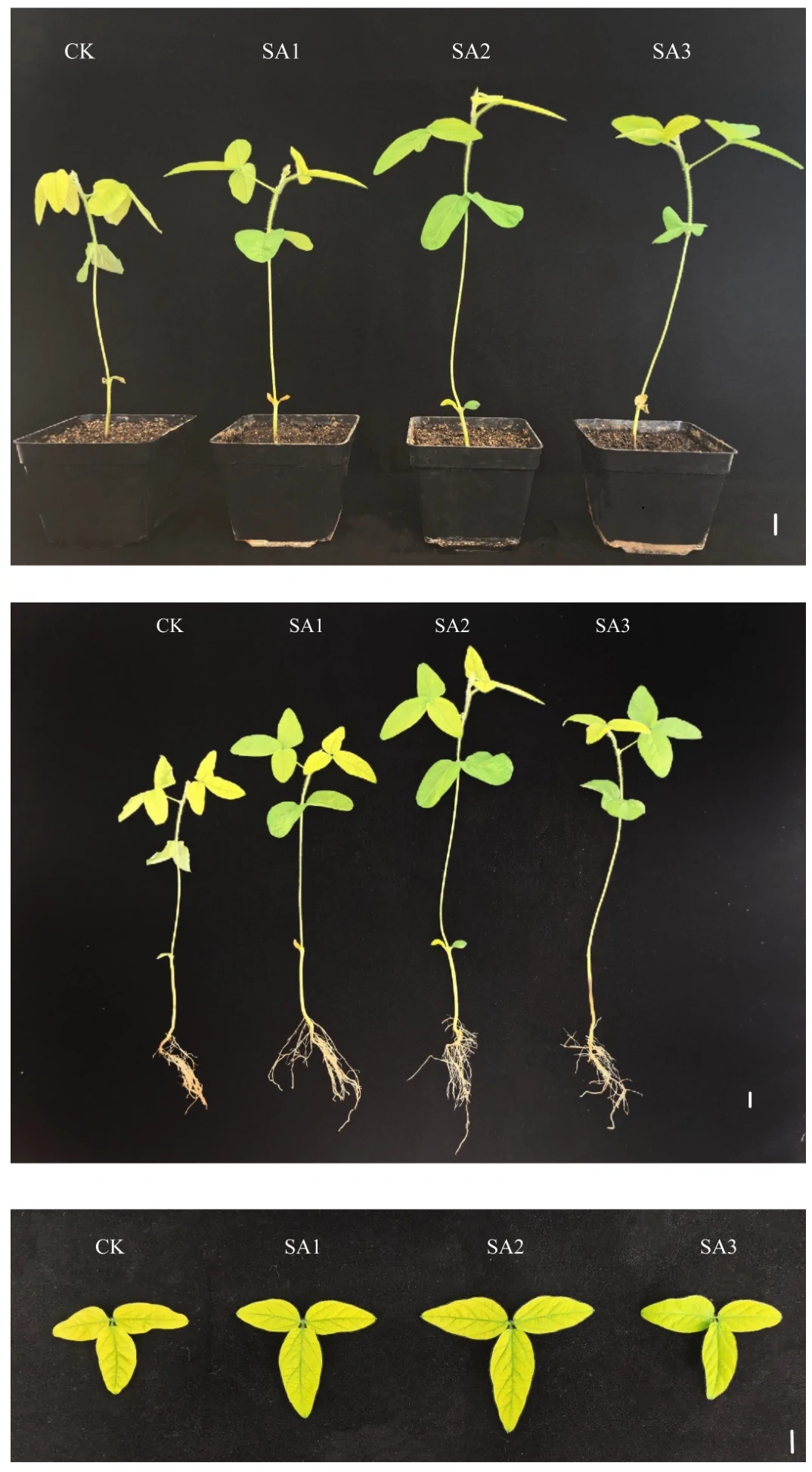
**Effects of nicotinamide treatment on phenotypes of soybeans under saline–alkali stress.** Scale bar = 1 cm. CK: saline–alkali-stressed control without nicotinamide treatment; SA1: 10 mg/L; SA2: 50 mg/L; SA3: 200 mg/L. Data are presented as mean ± SD (*n* = 3). Statistical significance was determined by one-way ANOVA followed by Tukey’s post hoc test (*p* < 0.05).

To investigate the effect of nicotinamide on saline–alkaline tolerance in soybean, a comprehensive set of growth- and physiology-related indicators was measured under salt-stressed conditions. The results showed that most agronomic traits—including plant height, stem and leaf fresh weight, root length, root fresh weight, stem and leaf dry weight, and root dry weight—all increased initially and then declined with increasing nicotinamide concentration, with the 50 mg/L treatment (SA2) generally exhibiting the most pronounced improvement effect on soybean growth and stress tolerance among all treatments relative to the salt-stressed CK group (Figure 1; Table 2).

**Figure 2:**
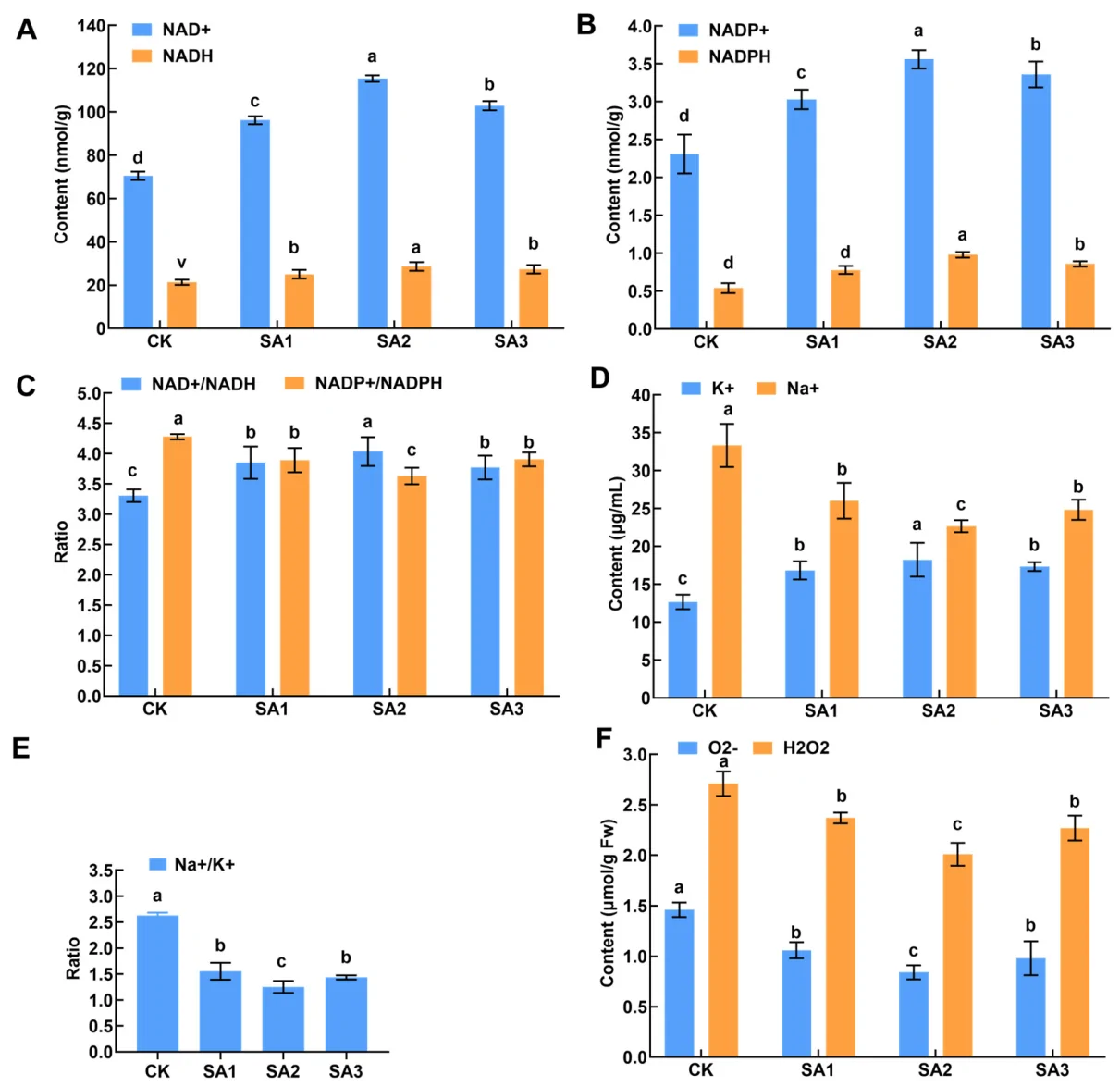
Effects of nicotinamide treatment on pyridine nucleotide contents, ion homeostasis, and ROS accumulation in soybeans under saline–alkali stress. (**A**) NAD^+^ and NADH contents. (**B**) NADP^+^ and NADPH contents. (**C**) NAD^+^/NADH, NADP^+^/NADPH ratios. (**D**) K^+^ and Na^+^ contents. (**E**) Na^+^/K^+^ ratio. (**F**) O_2_^−^· and H_2_O_2_ levels. CK: saline-alkali-stressed control without nicotinamide treatment; SA1: 10 mg/L; SA2: 50 mg/L; SA3: 200 mg/L. Different letters indicate significant differences among different nicotinamide concentration treatments. Data are presented as mean ± SD (*n* = 3). Statistical significance was determined by one-way ANOVA followed by Tukey’s post hoc test (*p* < 0.05).

As shown in Figure 2A–C, NAD^+^, NADH, NADP^+^, and NADPH contents, as well as the NAD^+^/NADH ratio, were significantly elevated under nicotinamide treatments compared with the CK group (*p* < 0.05), whereas the NADP^+^/NADPH ratio showed the opposite trend, indicating that nicotinamide application preferentially altered the pyridine nucleotide redox status in soybean leaves under saline–alkaline stress.

In addition, nicotinamide treatment exerted distinct effects on ion homeostasis. As illustrated in Figure 2D, K^+^ content increased significantly following nicotinamide treatment, while Na^+^ content decreased markedly compared with the CK group. Accordingly, the Na^+^/K^+^ ratio was also significantly reduced under nicotinamide treatments (Figure 2E). Notably, however, no significant difference was observed between the 10 mg/L (SA1) and 200 mg/L (SA3) treatment groups for most of the above indicators, suggesting that the promoting effect of nicotinamide on soybean saline–alkaline tolerance does not simply scale with concentration and may instead be subject to a physiological most effective state within salt-stressed conditions.

### 3.2. Nicotinamide Treatment Reduces ROS Accumulation in Soybeans Under Saline–Alkali Stress

Notably, saline–alkali stress caused ROS accumulation in soybean seedlings; however, nicotinamide application effectively reduced ROS levels. Specifically, nicotinamide treatment significantly reduced the superoxide anion (O^2−^·) content in soybean leaves (Figure 2F). Concomitantly, hydrogen peroxide (H_2_O_2_) content was also markedly decreased under nicotinamide treatments (Figure 2F), indicating that nicotinamide suppresses the accumulation of both primary and derived ROS under saline–alkali stress. Together with the reduced Na+ accumulation and increased K+ retention (Figure 2D,E), these results indicate that nicotinamide reduces ROS accumulation and improves ion homeostasis under saline–alkali stress.

### 3.3. Nicotinamide Application Enhances the Photosynthetic Pigment Content in Soybeans Under Saline–Alkali Stress

**Figure 3:**
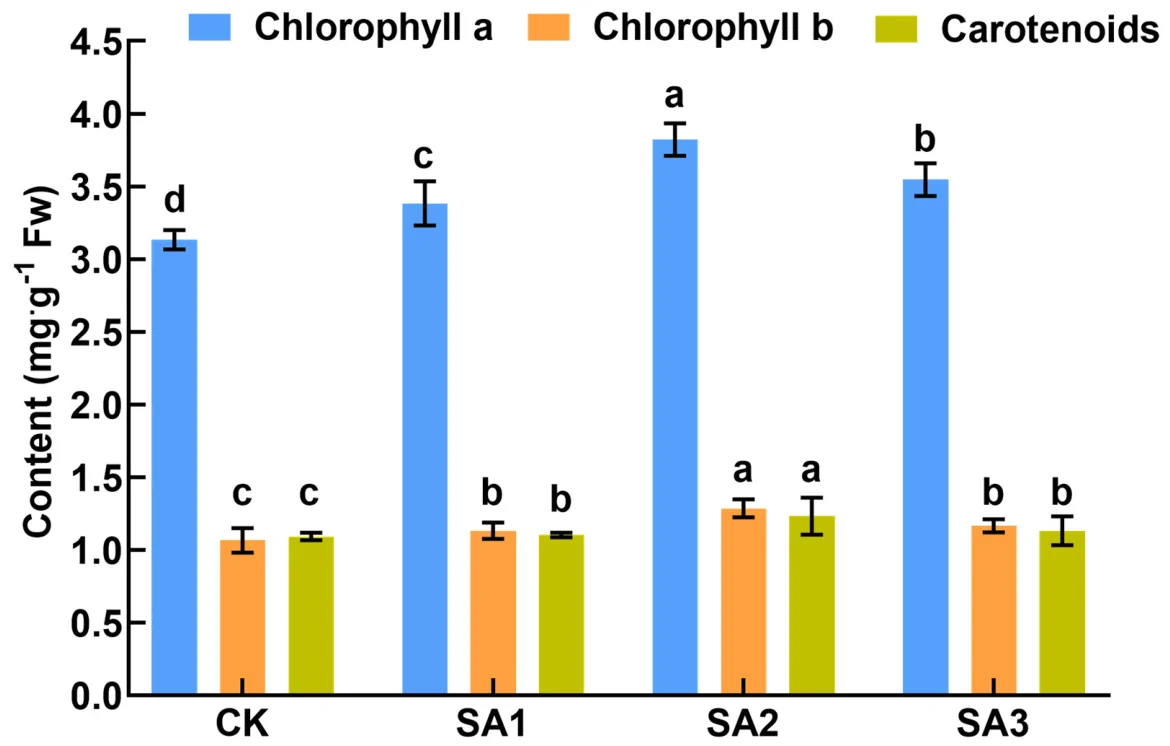
**Nicotinamide application enhances the photosynthetic pigment content of soybeans under saline–alkali stress.** CK: saline-alkali-stressed control without nicotinamide treatment; SA1: 10 mg/L; SA2: 50 mg/L; SA3: 200 mg/L. Different letters indicate significant differences among different nicotinamide concentration treatments. Data are presented as mean ± SD (*n* = 3). Statistical significance was determined by one-way ANOVA followed by Tukey’s post hoc test (*p* < 0.05).

Nicotinamide application exerted distinct effects on leaf photosynthetic pigment contents in soybean seedlings (Figure 3). Compared with the CK group, chlorophyll a content increased significantly under all nicotinamide treatments (*p* < 0.05). For chlorophyll b and total carotenoids, the 10, 50, and 200 mg/L nicotinamide treatments all significantly elevated their contents relative to the salt–alkali-stressed control. However, no significant differences in chlorophyll b or carotenoid contents were detected between the 10 mg/L (SA1) and 200 mg/L (SA3) groups, whereas chlorophyll a content in the 200 mg/L treatment was comparable to or slightly higher than that in the 10 mg/L treatment.

Collectively, these results indicate that nicotinamide application at all tested concentrations is associated with increased photosynthetic pigment contents; nevertheless, pigment levels did not further increase when the concentration was raised from 10 to 200 mg/L, suggesting a saturation effect rather than a continuous dose-dependent increase. It is worth noting that, based on comprehensive physiological performance, SA2 exhibited the most significant stress-alleviating effect (Figure 1 and Figure 2; Table 2). However, the response pattern of photosynthetic pigments alone did not exhibit a monotonic dose-dependent decline at higher concentrations. This discrepancy suggests that the inhibitory or less beneficial effects of supra-most effective nicotinamide concentrations on photosynthesis may be weaker than those on other physiological indices, such as ROS accumulation or ion homeostasis, and that the overall plant performance likely integrates multiple physiological pathways with distinct sensitivity to nicotinamide concentration.

### 3.4. Nicotinamide Administration Elevates the Osmoregulatory Product Contents and Antioxidant Enzyme Activities in Soybeans Under Saline–Alkali Stress

**Figure 4:**
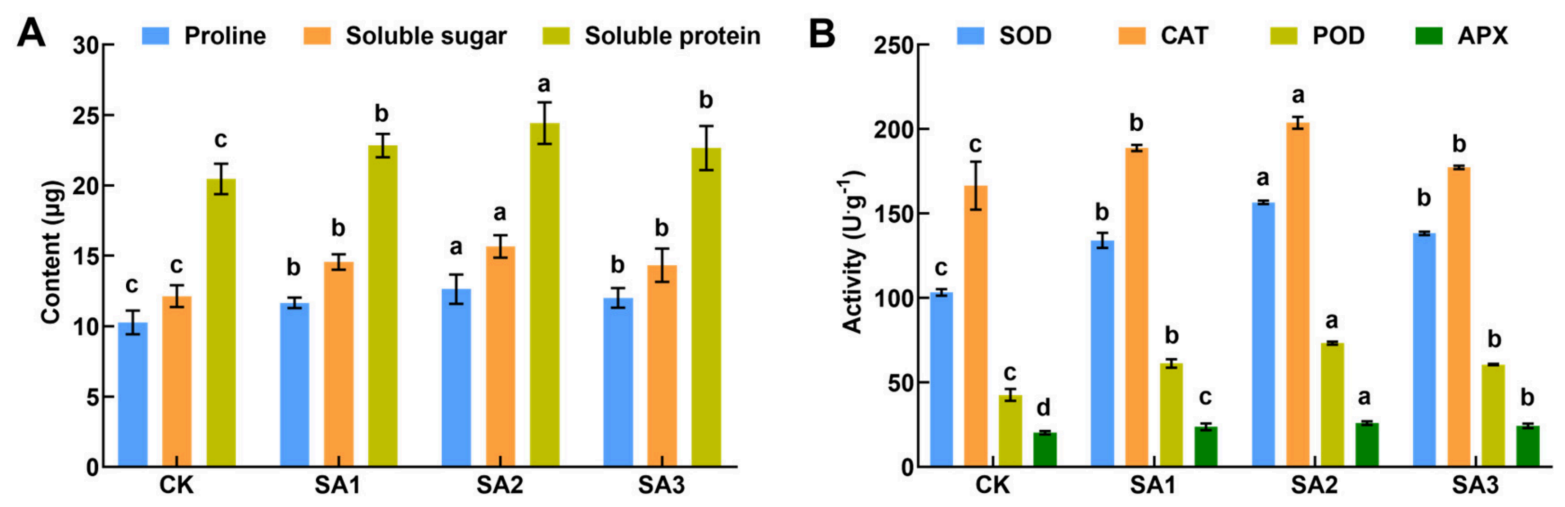
Nicotinamide administration elevates the contents of osmoregulatory products and antioxidant enzyme activities of soybeans under saline–alkali stress. (**A**) Osmoregulatory products. (**B**) Antioxidant enzyme activities. CK: saline–alkali-stressed control without nicotinamide treatment; SA1: 10 mg/L; SA2: 50 mg/L; SA3: 200 mg/L. Different letters indicate significant differences among different nicotinamide concentration treatments. Data are presented as mean ± SD (*n* = 3). Statistical significance was determined by one-way ANOVA followed by Tukey’s post hoc test (*p* < 0.05).

As shown in Figure 4, nicotinamide application increased the contents of osmoregulatory substances and the activities of antioxidant enzymes in soybean under saline–alkali stress. Under such stress, soluble protein, soluble sugar, and proline contents were all significantly increased following nicotinamide treatment compared with the CK group (Figure 4A). Similarly, the activities of antioxidant enzymes (SOD, CAT, POD, and APX) were also significantly enhanced relative to the control (Figure 4B).

Notably, among all detected osmoregulatory substances and antioxidant enzymes, the SA2 (50 mg/L) treatment consistently exhibited significantly higher levels than both the SA1 (10 mg/L) and SA3 (200 mg/L) treatments, as indicated by lowercase letter markings. Meanwhile, all indicators in the SA3 group were statistically superior to or comparable with those in the SA1 and CK groups. These results suggest that treatment with 50 mg/L nicotinamide confers the most prominent physiological improvement to alleviate saline–alkali damage in soybean.

### 3.5. Identification of DEGs

**Figure 5:**
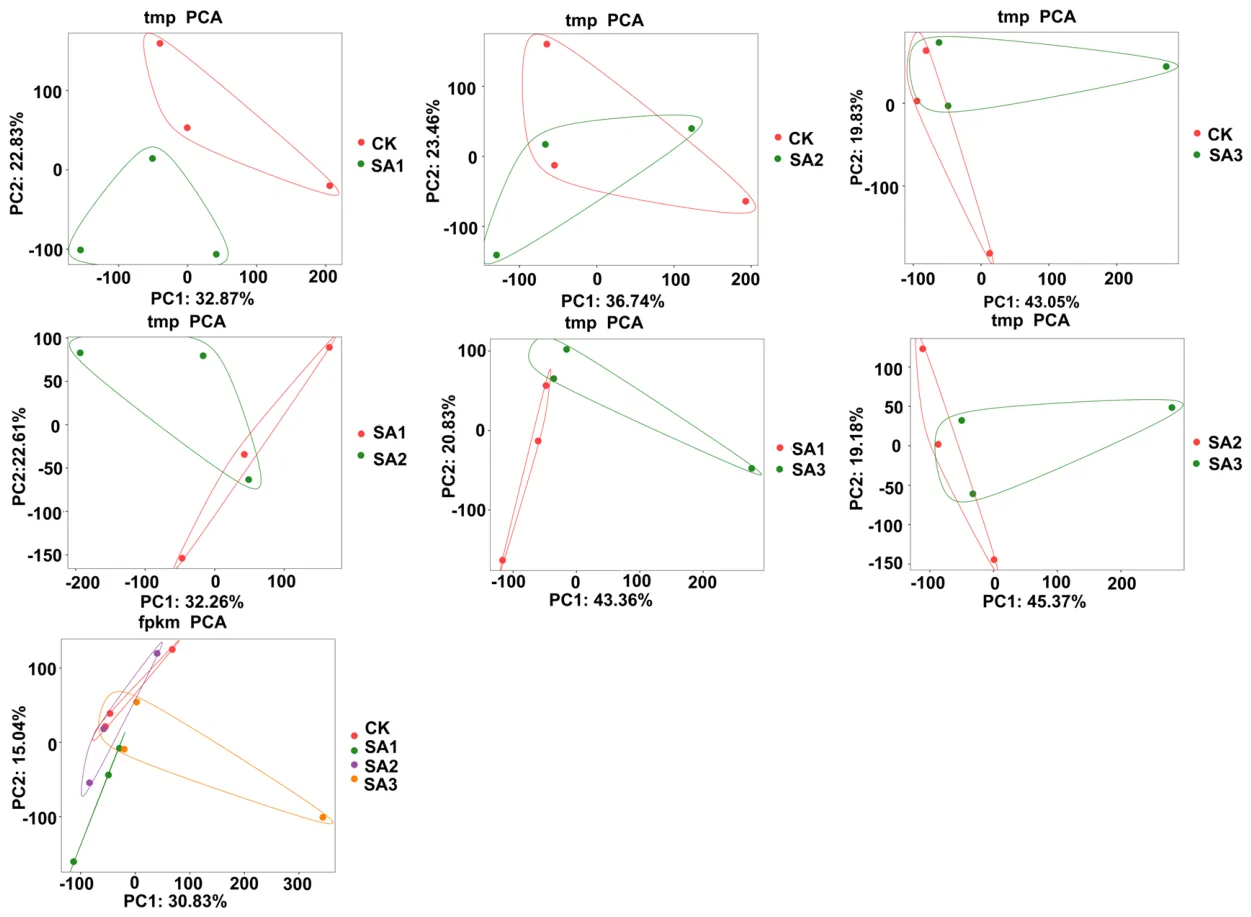
Principal component analysis (PCA) plots of sample quality control for control and nicotinamide administration. Error bars are not applicable to PCA.

**Figure 6:**
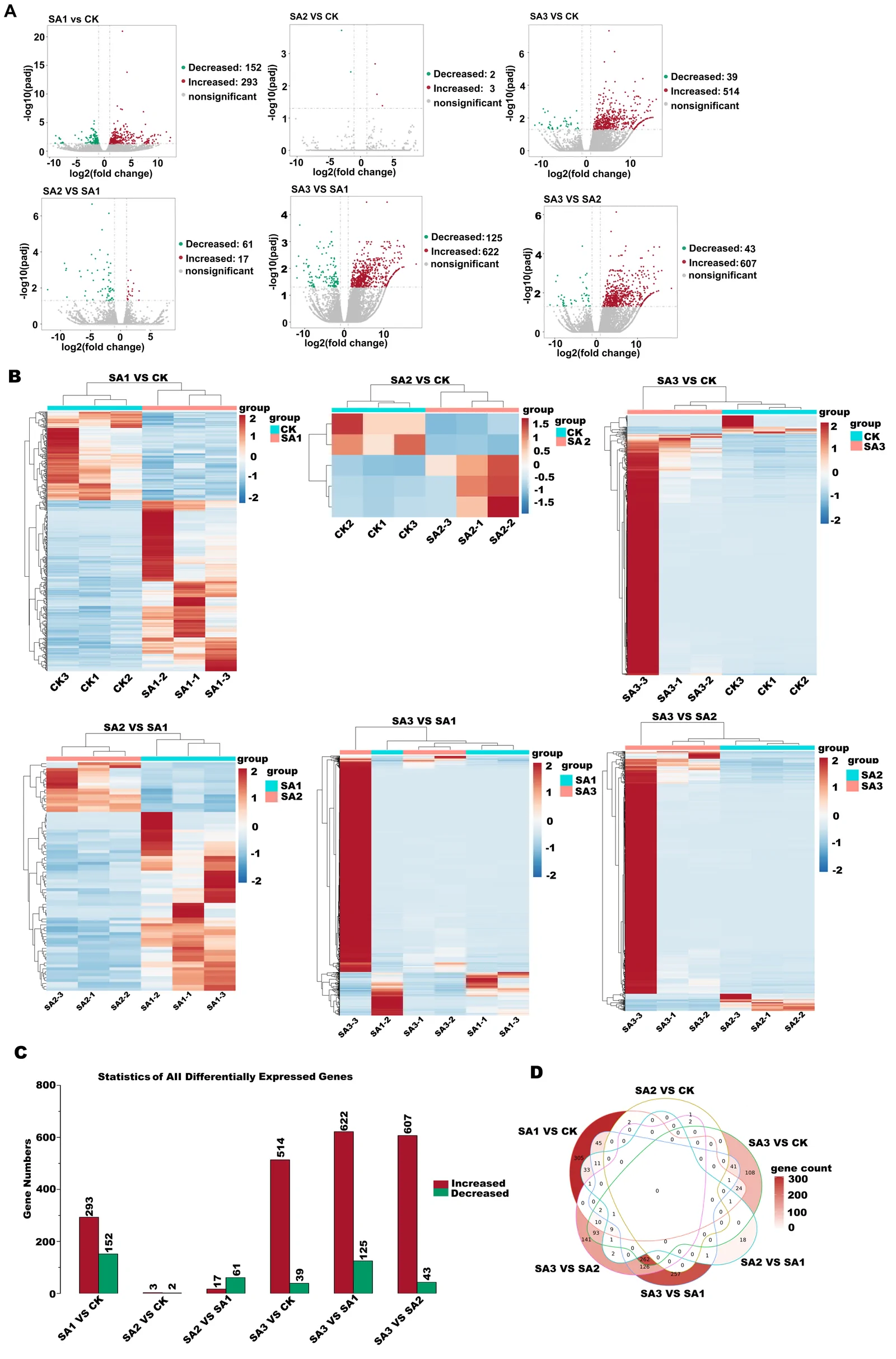

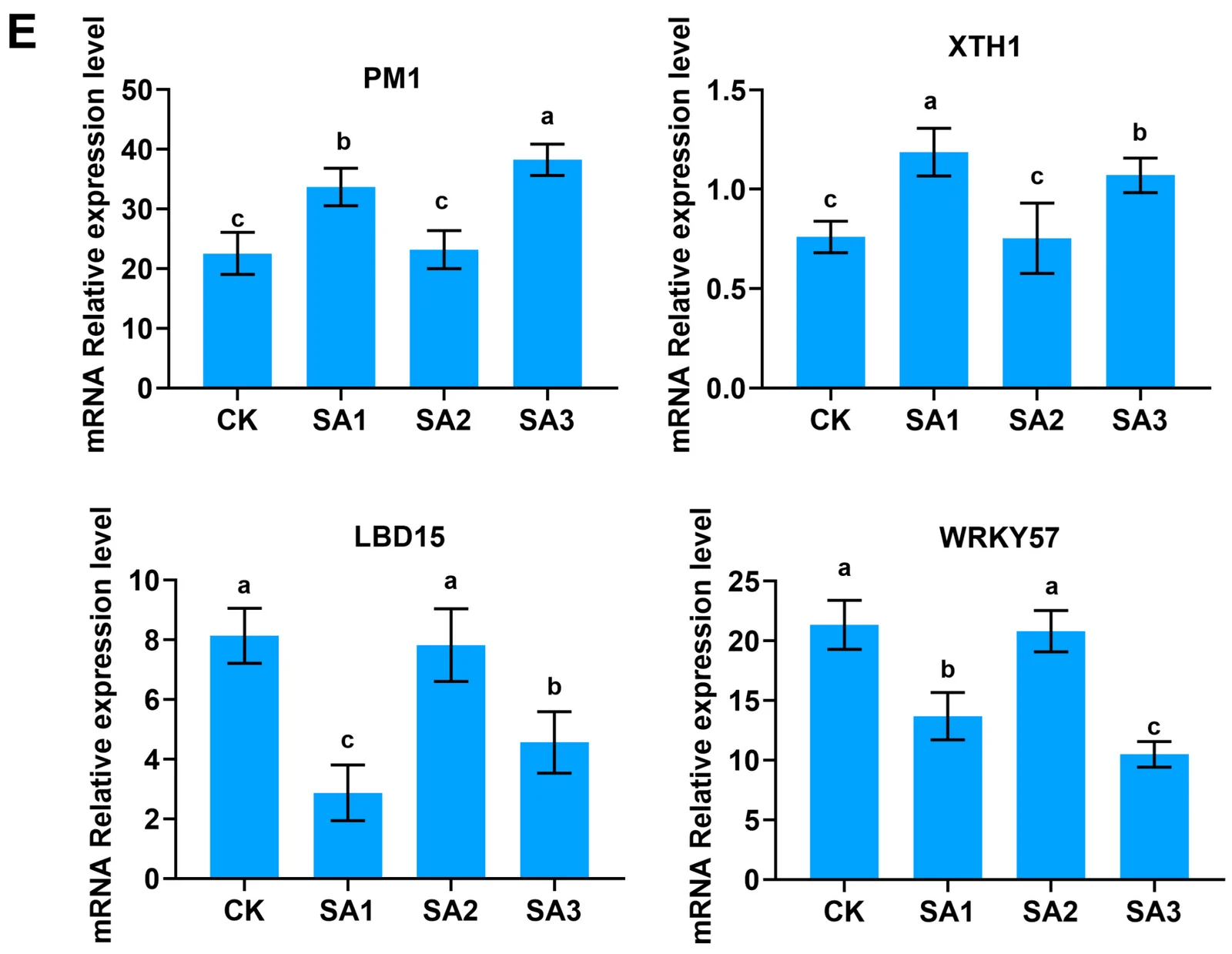
**Identification of differentially expressed genes (DEGs).** Volcano plot (**A**) and heatmap (**B**) of DEGs among SA1 vs. CK, SA2 vs. CK, SA3 vs. CK, SA2 vs. SA1, SA3 vs. SA1, and SA3 vs. SA2 groups. (**C**) Numbers of up- and downregulated DEGs in the six pairwise comparisons. (**D**) Venn diagram showing the overlaps of DEGs across all six pairwise comparison groups; no DEG was shared across all six groups. To screen core genes responding dynamically to nicotinamide concentrations deviating from the most effective dose, the overlapping set of the five comparisons excluding SA2 vs. CK was extracted, which yielded one shared DEG (*PM1*). Full DEG lists are provided in Supplementary Tables S2–S7. (**E**) RT-qPCR validation of *PM1*, *XTH1*, *WRKY57*, and *LBD15* expression in soybean leaves under saline–alkali stress. Data are presented as mean ± SD (*n* = 3). CK: saline–alkali-stressed control without nicotinamide treatment; SA1: 10 mg/L; SA2: 50 mg/L; SA3: 200 mg/L. Different letters indicate significant differences among treatments (one-way ANOVA followed by Tukey’s post hoc test, *p* < 0.05); RNA-seq DEGs were defined by DESeq2 (*p*.adj < 0.05, |log_2_FC| > 1).

After transcriptome sequencing, read quality was assessed, and all samples passed the QC thresholds. Principal component analysis (PCA) revealed that samples within each group exhibited good repeatability and clear inter-group separation (Figure 5), with no outlier samples detected, supporting the reliability of the sequencing data for subsequent differential expression analysis. Using the thresholds of adjusted *p*-value (*p*.adj) < 0.05 and |log_2_FC| > 1, we identified varying numbers of DEGs across six pairwise comparisons: 445 DEGs (293 upregulated and 152 downregulated) in SA1 vs. CK, five DEGs (three upregulated and two downregulated) in SA2 vs. CK, 553 DEGs (514 upregulated and 39 downregulated) in SA3 vs. CK, 78 DEGs (17 upregulated and 61 downregulated) in SA2 vs. SA1, 747 DEGs (622 upregulated and 125 downregulated) in SA3 vs. SA1, and 650 DEGs (607 upregulated and 43 downregulated) in SA3 vs. SA2 (Figure 6A–C; Supplementary Tables S2–S7).

Notably, the SA2 (50 mg/L) treatment exhibited far fewer DEGs relative to CK than SA1 and SA3. Because all comparisons were made against the stressed CK rather than a non-stressed control, the smaller number of DEGs in SA2 vs. CK indicates greater transcriptional similarity to the stressed control group, rather than recovery to a non-stressed state or direct evidence of stress alleviation. Conversely, the hundreds of DEGs detected in SA1 vs. CK and SA3 vs. CK indicate that the sub-effective and supra-effective concentrations were associated with more extensive transcriptional changes relative to the stressed control. Whether the limited transcriptional divergence of SA2 from the stressed CK is related to its superior physiological performance cannot be resolved by these comparisons alone and would require a non-stressed control group.

To characterize the transcriptional responses to different nicotinamide concentrations, we performed Venn overlap analyses at two levels addressing distinct biological questions (Figure 6D). First, global overlap analysis of all six comparison sets revealed no DEG shared across all treatments, indicating the absence of a universal transcriptional response common to all conditions. Second, to explore whether any genes responded consistently to nicotinamide concentrations deviating from the most physiologically effective dose, we performed a subset overlap analysis using the five comparisons, excluding SA2 vs. CK. This exclusion was exploratory rather than confirmatory, based on the physiological observation that SA2 exhibited the most pronounced stress-alleviating effects among the tested concentrations: SA1 vs. CK, SA3 vs. CK, SA2 vs. SA1, SA3 vs. SA1, and SA3 vs. SA2. This subset analysis identified one shared DEG, designated *PM1* (Figure 6D; Supplementary Tables S2–S7), suggesting that *PM1* is consistently responsive to nicotinamide under conditions deviating from the most physiologically effective concentration identified in this study. This observation tentatively identifies *PM1* as a potential candidate transcriptional reporter of dose-dependent transcriptional regulation; however, this finding is exploratory and requires functional validation.

To validate the reliability of the transcriptome data and further investigate the expression patterns of key genes across different nicotinamide concentrations, we performed RT-qPCR analysis on a panel of selected DEGs. These included *PM1*, the sole shared DEG identified in the subset Venn diagram analysis, as well as *XTH1* (upregulated) and *WRKY57* and *LBD15* (both downregulated) from the SA1 vs. CK comparison. As shown in Figure 6E and sequencing results (Supplementary Tables S2–S4), compared with CK under salt–alkali stress, the expression of *PM1* was significantly increased in the SA1 and SA3 groups (SA1 vs. CK: log_2_FC = 5.6448, *p*.adj = 0.0166; SA3 vs. CK: log_2_FC = 11.8675, *p*.adj = 0.0004; SA2 vs. CK: log_2_FC = −3.4375, *p*.adj = 1). RT qPCR validation (*p* = 0.0151 for SA1 vs. CK, *p* = 0.0035 for SA3 vs. CK, and *p* = 0.8284 for SA2 vs. CK) verified that *PM1* exhibits a dose-dependent expression pattern that may be useful for distinguishing non-most effective nicotinamide concentrations; however, further functional validation—such as gene overexpression, knockout, or transient expression studies—is required to confirm its biological role. For *XTH1*, transcript abundance was further upregulated under SA1 conditions, whereas SA2 treatment effectively suppressed its expression to levels comparable to the salt–alkali-stressed control. Conversely, *WRKY57* and *LBD15*—both transcriptionally repressed under stress—exhibited pronounced upregulation specifically in the SA2 group, indicating that 50 mg/L nicotinamide treatment effectively relieves stress-induced transcriptional repression of these defense-related genes while maintaining transcriptional consistency with stressed CK.

### 3.6. GO and KEGG Enrichment Analysis

**Figure 7:**
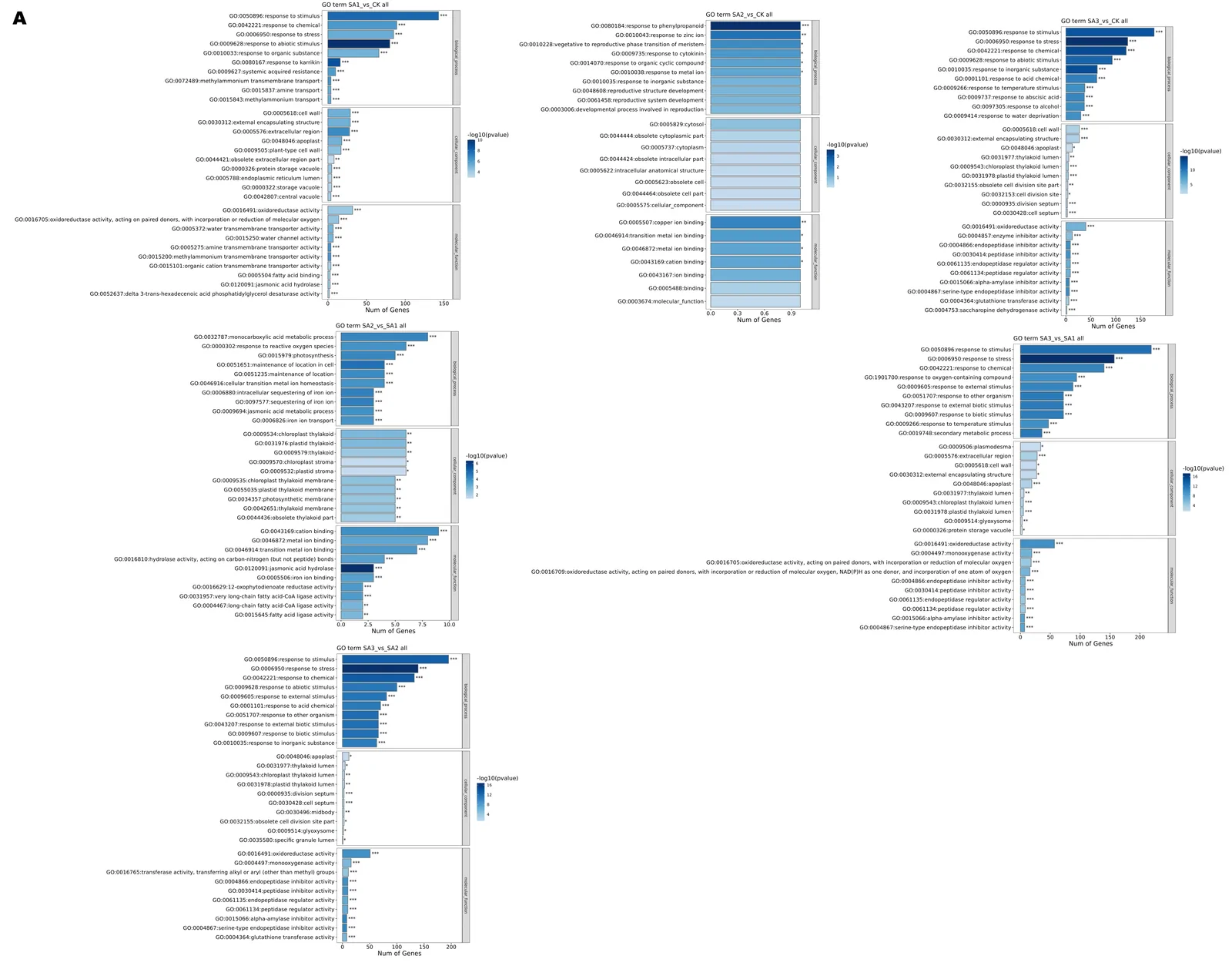

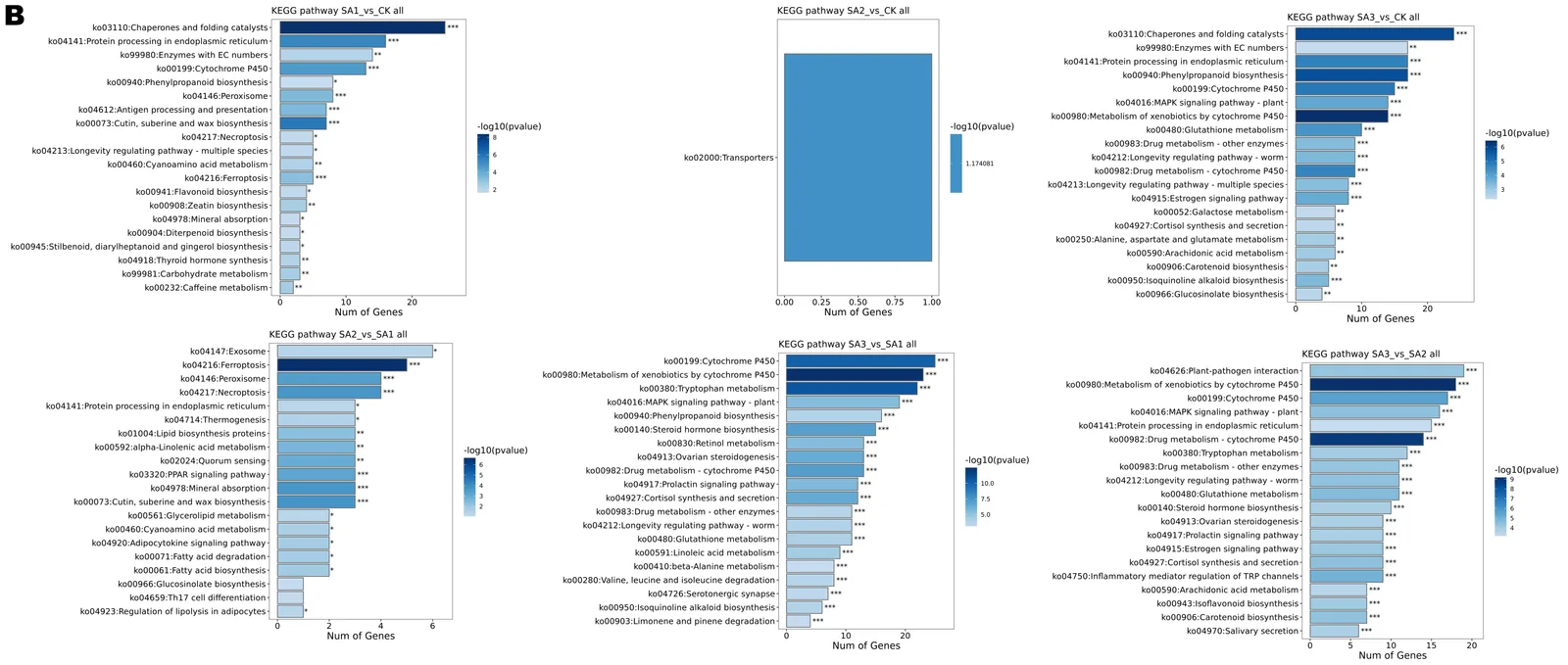

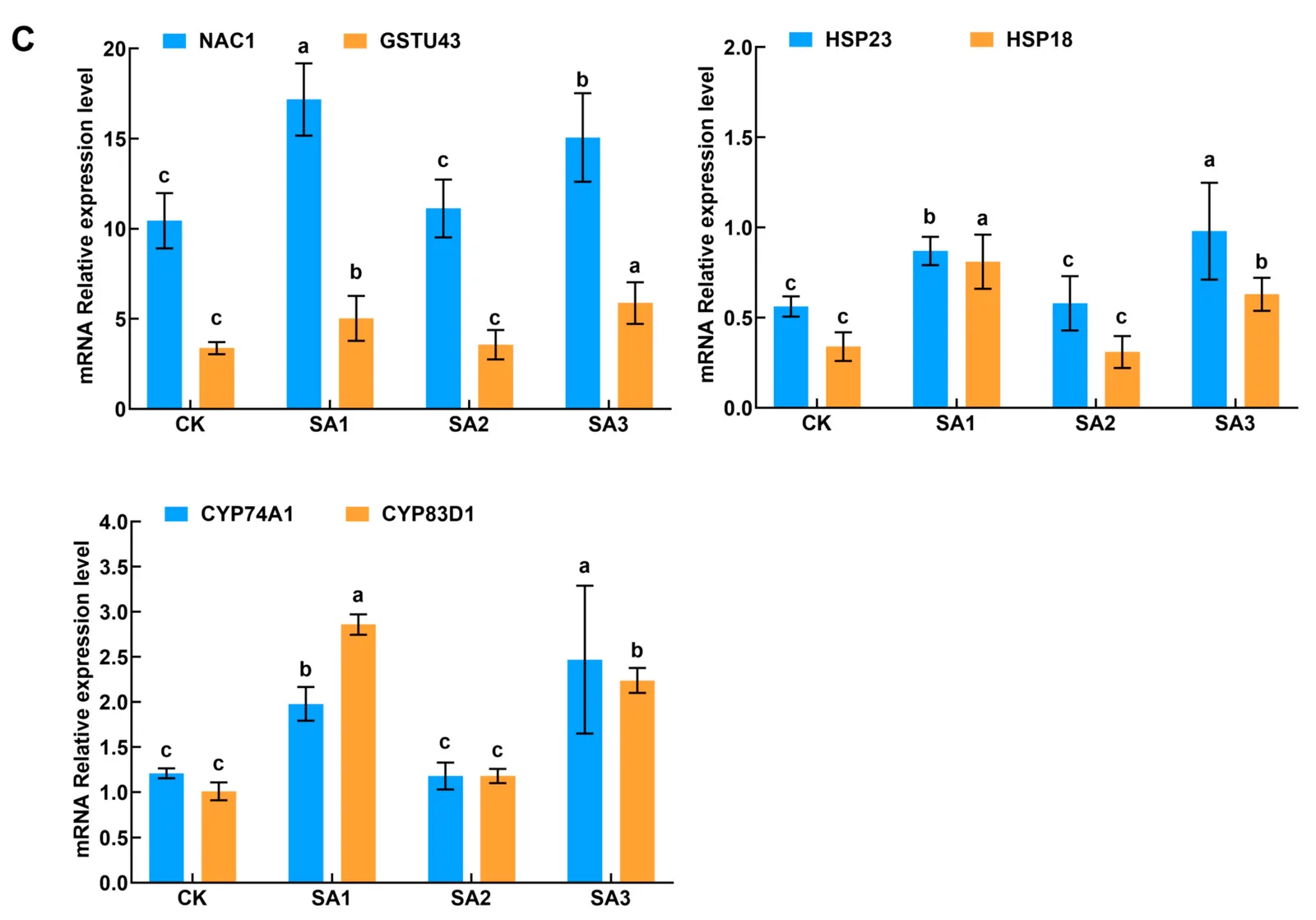
Gene Ontology (GO) and Kyoto Encyclopedia of Genes and Genomes (KEGG) enrichment analysis of differentially expressed genes (DEGs). (**A**) GO terms. (**B**) KEGG pathways. (**C**) RT-qPCR validation of key genes enriched in core KEGG pathways: *CYP74A1*, *CYP83D1*, *HSP23*, *HSP18*, *NAC1*, and *GSTU43*. Data are presented as mean ± SD (*n* = 3). CK: saline–alkali-stressed control without nicotinamide treatment; SA1: 10 mg/L; SA2: 50 mg/L; SA3: 200 mg/L. Different letters indicate significant differences among treatments (one-way ANOVA followed by Tukey’s post hoc test, *p* < 0.05). Enrichment significance was defined as *p*.adj < 0.05 by clusterProfiler.

To investigate the biological functions of the DEGs identified in the six pairwise comparisons, GO and KEGG pathway enrichment analyses were performed using the soybean genome as the background gene set (Figure 7A,B).

For DEGs identified in SA1 vs. CK, the enriched GO terms included GO:0050896 (response to stimulus, GeneRatio = 143/262, *p*.adj = 7.12 × 10^6^), GO:0005618 (cell wall, GeneRatio = 29/262, *p*.adj = 0.00094), and GO:0016491 (oxidoreductase activity, GeneRatio = 32/262, *p*.adj = 0.004096), whereas the enriched KEGG pathways mainly comprised ko03110 (chaperones and folding catalysts, GeneRatio = 25/199, *p*.adj = 5.83 × 10^7^), ko04141 (protein processing in endoplasmic reticulum, GeneRatio = 16/199, *p*.adj = 0.000194), and ko00199 (cytochrome P450, GeneRatio = 13/199, *p*.adj = 0.000693).

For DEGs identified in SA2 vs. CK, the only significantly enriched GO term was GO:0080184 (response to phenylpropanoid, GeneRatio = 6/24,388, *p*.adj = 0.009594); no KEGG pathway reached the significance threshold. The top-ranked but non-significant terms, including GO:0005829 (cytosol, GeneRatio = 3473/24,388, *p*.adj = 0.251344), GO:0005507 (copper ion binding, GeneRatio = 204/24,388, *p*.adj = 0.108742), and ko02000 (transporters, GeneRatio = 1458/21,769, *p*.adj = 0.06697), are reported as descriptive trends only and are not considered enriched.

For DEGs identified in SA3 vs. CK, the enriched GO terms included GO:0050896 (response to stimulus, GeneRatio = 177/308, *p*.adj = 2.87 × 10^10^), GO:0005618 (cell wall, GeneRatio = 27/308, *p*.adj = 0.012298), and GO:0016491 (oxidoreductase activity, GeneRatio = 40/308, *p*.adj = 9.22 × 10^5^), and the enriched KEGG pathways mainly comprised ko03110 (chaperones and folding catalysts, GeneRatio = 24/249, *p*.adj = 0.000113), ko04141 (protein processing in endoplasmic reticulum, GeneRatio = 17/249, *p*.adj = 0.000771), and ko00199 (cytochrome P450, GeneRatio = 15/249, *p*.adj = 0.000638).

For DEGs identified in SA2 vs. SA1, they were primarily enriched in GO terms such as GO:0032787 (monocarboxylic acid metabolic process, GeneRatio = 8/51, *p*.adj = 0.010189), GO:0009534 (chloroplast thylakoid, GeneRatio = 6/51, *p*.adj = 0.029499), and GO:0043169 (cation binding, GeneRatio = 9/51, *p*.adj = 0.010487), as well as the significantly enriched KEGG pathways ko04216 (ferroptosis, GeneRatio = 5/39, *p*.adj = 1.29 × 10^5^) and ko04146 (peroxisome, GeneRatio = 4/39, *p*.adj = 0.004140). ko04141 (protein processing in endoplasmic reticulum, GeneRatio = 3/39, *p*.adj = 0.152100) ranked among the top KEGG results but exceeded the significance threshold and is, therefore, described as a non-significant trend only.

For DEGs identified in SA3 vs. SA1, the significantly enriched GO terms included GO:0050896 (response to stimulus, GeneRatio = 219/399, *p*.adj = 8.18 × 10^10^) and GO:0016491 (oxidoreductase activity, GeneRatio = 57/399, *p*.adj = 6.88 × 10^8^), whereas GO:0009506 (plasmodesma, GeneRatio = 34/399, *p*.adj = 0.170760) was among the top-ranked terms but did not reach significance. The significantly enriched KEGG pathways mainly comprised ko00199 (cytochrome P450, GeneRatio = 25/318, *p*.adj = 4.49 × 10^9^), ko00980 (metabolism of xenobiotics by cytochrome P450, GeneRatio = 23/318, *p*.adj = 6.46 × 10^11^), and ko04016 (MAPK signaling pathway-plant, GeneRatio = 19/318, *p*.adj = 7.86 × 10^5^).

For DEGs identified in SA3 vs. SA2, the significantly enriched GO terms included GO:0050896 (response to stimulus, GeneRatio = 195/342, *p*.adj = 7.47 × 10^11^) and GO:0016491 (oxidoreductase activity, GeneRatio = 51/342, *p*.adj = 9.12 × 10^8^), whereas GO:0048046 (apoplast, GeneRatio = 12/342, *p*.adj = 0.231624) was among the top-ranked terms but did not reach significance. The significantly enriched KEGG pathways mainly comprised ko00980 (metabolism of xenobiotics by cytochrome P450, GeneRatio = 18/275, *p*.adj = 1.32 × 10^7^), ko00199 (cytochrome P450, GeneRatio = 17/275, *p*.adj = 0.000153), and ko04016 (MAPK signaling pathway-plant, GeneRatio = 16/275, *p*.adj = 0.000824).

To confirm the validity of the key enriched pathways, we selected six representative genes from the cytochrome P450 detoxification, protein folding and ER processing, and ROS scavenging pathways for RT-qPCR verification (Figure 7C). The results showed that two cytochrome P450 family genes (*CYP74A1, CYP83D1*) and two HSP chaperone genes *(HSP23, HSP18*) were significantly upregulated in the SA1 and SA3 groups, but showed no significant difference between the SA2 and salt–alkali-stressed CK groups. In addition, the ROS scavenging-related genes *NAC1* and *GSTU43* were strongly induced under the sub-most effective and supra-most effective nicotinamide treatments, while their expression was similar to that in the salt–alkali-stressed CK under the most effective 50 mg/L treatment. The consistent expression patterns of pathway-specific functional genes further corroborated the transcriptome enrichment results. Collectively, these findings indicate that non-most effective nicotinamide concentrations are associated with marked transcriptional changes in genes annotated to ER protein processing, detoxification, and oxidative stress-related pathways, whereas 50 mg/L shows limited transcriptional divergence from the salt–alkali-stressed control, i.e., a transcriptomic profile more similar to the stressed control. Whether these transcriptional changes reflect actual ER stress, detoxification responses, or oxidative stress at the cellular level requires direct experimental validation.

## 4. Discussion

Salt–alkali stress ranks among the most detrimental abiotic stresses, adversely affecting soybean productivity and quality [25]. Accumulating evidence demonstrates that enhancing endogenous nicotinamide accumulation effectively promotes plant growth and development under abiotic stress [14,26]. Therefore, this study investigated the role of nicotinamide in soybean salt–alkaline tolerance. Physiological measurements demonstrated that nicotinamide treatment enhances salt–alkaline tolerance in soybeans, with 50 mg/L showing the most pronounced physiological improvements. Transcriptomic analysis was employed to compare the transcriptional landscapes across different nicotinamide concentrations. Notably, transcriptomic analysis revealed that the 50 mg/L treatment (SA2) induced only five differentially expressed genes (DEGs) compared with the stressed control (CK), whereas the sub-most effective (SA1, 10 mg/L) and supra-most effective (SA3, 200 mg/L) concentrations induced hundreds of DEGs (445 and 553, respectively). This pattern is consistent with the interpretation that the number of DEGs reflects the extent of transcriptional disturbance rather than the efficacy of stress alleviation: the 50 mg/L concentration is associated with physiological improvements and exhibits the fewest DEGs relative to the salt–alkali-stressed CK, raising the possibility that effective stress alleviation may be associated with limited transcriptional reprogramming relative to the stressed control. Similar phenomena have been observed in other plant stress studies, where moderate stress or effective treatment conditions often result in fewer transcriptional changes than severe stress or excessive treatments [27], supporting our interpretation. Consistent with this notion, Wang et al. (2025) demonstrated that maize roots under moderate drought exhibited only 903 DEGs, whereas severe drought triggered 5306 DEGs [28]. Xu et al. (2023) reported that a drought-tolerant soybean cultivar showed merely 479 DEGs under mild drought, in contrast to 3317 DEGs in a drought-sensitive cultivar [29]. Li et al. (2022) further observed that mild drought stress exclusively enriched osmotic adjustment pathways, whereas defense pathways were activated mainly by severe drought [30]. Collectively, these studies in maize and other species support the generality of this transcriptional dose–response pattern across different crops.

Consistent with previous reports [31,32,33], salt–alkali stress induced substantial Na^+^ accumulation and K^+^ depletion in soybean seedlings, whereas nicotinamide application significantly improved ion homeostasis by enhancing K^+^ retention and reducing Na content and the Na^+^/K^+^ ratio. Furthermore, nicotinamide treatments significantly elevated photosynthetic pigment contents (chlorophyll a, chlorophyll b, and carotenoids), which are known to be impaired under salt–alkali stress through ion toxicity and oxidative damage [34,35]. In addition, nicotinamide promoted the accumulation of key osmotic regulatory substances, including proline, soluble sugars, and soluble proteins, which are recognized as important indicators of plant adaptive capacity and stress response [36,37]. The activities of enzymatic antioxidants (SOD, POD, CAT, and APX) were also markedly enhanced following nicotinamide application, consistent with their central roles in plant defense mechanisms [38] and with previous observations in barley under water deficit [39]. Collectively, these physiological results demonstrate that nicotinamide treatment confers multi-faceted protection against salt–alkali stress in soybean, with 50 mg/L showing the most pronounced improvements.

To elucidate the transcriptional basis underlying the efficacy of 50 mg/L nicotinamide, we performed a layered Venn overlap analysis distinguishing between global transcriptional responses and dose-dependent dynamics. Global overlap analysis of all six comparison groups revealed no shared DEGs, indicating the absence of a universal transcriptional response. Subsequently, subset overlap analysis focusing on five comparisons representing transcriptional responses under the non-most effective conditions (SA1 vs. CK, SA3 vs. CK, SA2 vs. SA1, SA3 vs. SA1, and SA3 vs. SA2) identified a single shared DEG, *PM1*. This gene was excluded from the SA2 vs. CK comparison as part of an exploratory subset analysis because it was not differentially expressed under the 50 mg/L treatment. Based on this exploratory approach, we tentatively propose that *PM1* may act as a candidate transcriptional reporter of nicotinamide dose deviation; however, this interpretation is hypothesis-generating rather than confirmatory, and requires independent experimental validation: its expression is consistently perturbed when the concentration deviates from the physiologically effective dose with limited transcriptional disturbance but is similar to the stressed control under most effective conditions, suggesting that it is not a direct target of nicotinamide. RT-qPCR confirmed that *PM1* was significantly induced under SA1 and SA3, but returned to levels comparable to the salt–alkali-stressed CK specifically in SA2 (Figure 6E). This pattern differed from the expression patterns of *XTH1* (upregulated in SA1, suppressed in SA2) and *WRKY57*/*LBD15* (repressed under stress, derepressed relative to the stressed CK in SA2), suggesting that *PM1* is not a general stress-responsive gene but a dose-sensitive marker. It should be noted that, because all experimental groups were subjected to saline–alkaline stress, we cannot rule out the possibility that *PM1* also responds to salt stress per se; however, its dose-dependent transcriptional pattern clearly indicates a primary responsiveness to nicotinamide concentration. Its bidirectional response to variations in concentration, with expression matching the salt–alkali-stressed CK only at 50 mg/L, suggests that it may have potential utility as a dose-sensitive indicator, though this possibility requires further functional validation.

PM1 belongs to the LEA_1 subfamily of LEA proteins, which enhance plant tolerance to abiotic stresses (e.g., drought and salinity) through membrane stabilization, metal ion chelation, and oxidative damage mitigation [40]. Liu et al. demonstrated that the PM1 protein binds various metal ions, including Fe^3+^, Ni^2+^, Cu^2+^, and Zn^3+^, thereby exerting crucial protective effects against oxidative damage and ion toxicity under abiotic stress [41]. Additionally, Shih et al. reported that interactions between the soybean PM1 protein and POPC lipids maintain the liquid-crystalline phase over an extended temperature range in the desiccated state [42]. Previous studies have also shown that, in addition to maintaining cytoplasmic ion homeostasis, PM1 proteins stabilize and protect other proteins under abiotic stress by assembling an oligomeric molecular shield around target biomolecules [43]. Consistent with these established functions, our observation that *PM1* expression was comparable to that of the salt–alkali-stressed CK specifically under the 50 mg/L treatment, but was altered under both lower and higher concentrations, suggests that this dose-dependent expression pattern may reflect transcriptional adjustment to nicotinamide concentration and could serve as a candidate transcriptional reporter, though it does not indicate stress alleviation per se. The fact that *PM1* responds to both under- and over-dosing further supports its potential as a dose-sensitive indicator, but this interpretation remains correlational and requires functional validation. RT-qPCR validation confirmed that the transcript levels of representative genes from the key enriched pathways (*CYP74A1*, *CYP83D1*, *HSP23*, *HSP18*, *NAC1*, and *GSTU43*) were significantly elevated under SA1 and SA3, whereas their transcript abundance under SA2 was comparable to that of the stressed CK (Figure 7C). This consistency supports our transcriptome findings and suggests that non-most effective doses were associated with transcriptional changes in pathways related to ER stress, detoxification, and oxidative responses, whereas 50 mg/L nicotinamide was associated with markedly fewer transcriptional changes and a transcriptomic profile more similar to the stressed CK. It should be noted that these observations represent changes in transcript abundance; the corresponding protein activities and functional consequences remain to be experimentally verified. The simultaneous downregulation of these functionally diverse genes under SA2 treatment suggests the possibility of a common upstream regulatory influence, potentially involving NAD^+^/NADH redox balance or ER stress sensing; however, this interpretation remains speculative and requires direct experimental testing.

Nicotinamide is a key precursor of NAD^+^ and NADP^+^ [44], and its exogenous application directly elevated the cellular NAD^+^/NADH ratio, whereas the NADP^+^/NADPH ratio showed a contrasting tendency (Figure 2A–C). This coenzyme replenishment may have two immediate consequences. First, enhanced NADPH supply might theoretically fuel the glutathione–ascorbate cycle, which could contribute to the antioxidant defense system and reducing ROS accumulation, consistent with our observed increases in antioxidant enzyme activities (Figure 4B) and decreases in H_2_O_2_ and O^2−^· levels (Figure 2F). Second, optimization of NAD^+^/NADH status relative to the salt–alkali-stressed CK could potentially improve endoplasmic reticulum (ER) redox homeostasis, possibly alleviating stress-induced ER redox perturbations and reducing misfolded protein accumulation. However, these proposed links between coenzyme changes and downstream physiological outcomes require direct experimental validation. This is consistent with the enrichment of DEGs in the ko04141 (protein processing in ER) and ko03110 (chaperones and folding catalysts) pathways, particularly under the sub-most effective SA1 and supra-most effective SA3 conditions, where enrichment of these ER-related pathways was most pronounced. The ER protein processing pathway (ko04141) ensures quality control of nascent polypeptides, while the chaperone and folding catalyst pathway (ko03110) mediates protein folding and refolding. Their concurrent enrichment suggests a possible role for nicotinamide in ameliorating ER stress, potentially through promoting proper protein maturation and preventing aggregation [45]. Collectively, the coenzyme-mediated mechanisms discussed above are inferred from changes in NAD^+^/NADH and NADP^+^/NADPH ratios and from KEGG enrichment of related pathways. Direct measurements of glutathione, ascorbate, ER stress markers, UPR markers, and protein folding status are needed to confirm these hypotheses. This proposed ER-protective role, if confirmed, would be particularly critical under salt–alkali stress, which is known to disrupt ER homeostasis and trigger the unfolded protein response (UPR) [46,47].

Third, notably, enrichment of the cytochrome P450 pathway (ko00199) suggests a possible involvement of cytochrome P450 in priming cellular detoxification systems. Cytochrome P450 monooxygenases are central to xenobiotic metabolism and endogenous signaling molecule biosynthesis; consequently, their enrichment in DEGs may tentatively suggest a role in chemical defense [48,49,50]. However, this interpretation is based solely on pathway enrichment and requires functional validation through enzymatic activity assays or genetic perturbation studies. We tentatively speculate that P450-mediated detoxification may operate concurrently with ER stress alleviation pathways, potentially contributing to a coordinated protective response. One arm may mitigate stress-disrupted protein status (through ER processing and chaperones), while the other may enhance chemical defense (through P450-mediated ROS scavenging and toxin metabolism). However, direct experimental evidence—such as enzymatic activity assays or genetic perturbation studies—is required to confirm this hypothesis. Crucially, *PM1* is not a component of these specific pathways; rather, its expression comparable to the salt–alkali-stressed CK under the most effective 50 mg/L treatment serves as an integrative readout of successful stress recovery, reflecting coordinated amelioration of stress-induced disruptions to protein status, detoxification and cellular repair. This hierarchical model explains why SA2 exhibits the fewest DEGs and a transcriptomic profile most similar to the stressed CK: if a coordinated network of ER protection, P450-mediated detoxification, and *PM1* homeostasis is established, the plant no longer requires large-scale transcriptional reprogramming and can maintain stress tolerance through fine-tuned steady-state regulation under sustained saline–alkali stress conditions. This model, however, remains hypothetical and requires functional validation.

In summary, we propose a working hypothesis that nicotinamide-mediated salt–alkaline tolerance may involve coordinated, multi-layered processes: (i) potential amelioration of stress-disrupted coenzyme balance (NAD^+^/NADH and NADP^+^/NADPH), suggested by the coenzyme measurements; (ii) possible involvement of ER stress alleviation pathways, based on the enrichment of genes annotated to protein processing in the ER (ko04141) and chaperone/folding catalysts (ko03110); (iii) potential involvement of P450-mediated chemical detoxification (ko00199), based on the enrichment of DEGs in this pathway; and (iv) the observation that *PM1* expression was restored to a level comparable to the stressed CK specifically under SA2, which may be associated with effective alleviation of stress-induced cellular perturbations. This integrated framework provides a working hypothesis for the potential molecular processes underlying nicotinamide’s protective effects and may serve as a reference for future studies on stress tolerance enhancement in soybean.

Although this study elucidates the role of and potential mechanisms by which nicotinamide regulates salt–alkaline tolerance in soybeans, several limitations of this study should be acknowledged before interpreting the findings. First, the candidate gene *PM1* and the enriched KEGG pathways warrant functional validation through targeted experiments, such as gene overexpression/knockout or pharmacological inhibition, in future studies. Without such validation, the transcriptional observations reported here should not be interpreted as direct evidence of pathway activation or gene function. Second, and equally critically, the experimental design lacks both a non-stressed control (without salt stress) and a nicotinamide-only control (without salt stress). Consequently, we cannot distinguish whether the observed transcriptional changes are driven by salt stress itself, by nicotinamide under normal conditions, or by their interaction. This also precludes any definitive conclusion that SA2 “restores” transcription to a non-stressed state; rather, our conclusions are strictly confined to comparisons with the saline–alkali-stressed control. Third, the precise molecular mechanism by which *PM1* exerts its protective function in nicotinamide-mediated stress alleviation warrants further exploration, particularly regarding its interactions with other stress-responsive proteins and signaling components. Fourth, because our findings are based on a single soybean cultivar grown under controlled greenhouse conditions, their generalizability to other cultivars or field environments remains to be determined. Fifth, *GAPDH* was used as the sole reference gene for RT-qPCR normalization in this study. Although *GAPDH* has been validated as a stable reference gene in soybean under various abiotic stress conditions, including salinity and alkaline stress, in previous studies [6,8], we acknowledge that using multiple reference genes would further strengthen the reliability of the expression patterns. Future studies will incorporate additional internal controls (e.g., *Actin, EF-1α*, or *TUBA*) to validate the dose-dependent expression patterns reported here. Sixth, the proposed mechanisms involving the NADPH–glutathione–ascorbate cycle and ER redox homeostasis are inferred from NAD^+^/NADH/NADP^+^/NADPH measurements and KEGG enrichment analyses, rather than from direct measurements of glutathione, ascorbate, ER stress markers, UPR markers, or protein misfolding. Therefore, these interpretations remain hypothetical and require direct experimental validation in future studies. Future studies incorporating non-stressed and nicotinamide-only controls will be essential to establish a complete transcriptional baseline and to validate the effects of nicotinamide under both stress and non-stress conditions.

## 5. Conclusions

This study demonstrates that exogenous nicotinamide application enhances salt–alkaline tolerance in soybeans, with 50 mg/L showing the most pronounced physiological improvements among all tested concentrations. Transcriptome analysis revealed that 50 mg/L induced limited transcriptional perturbation (only five DEGs), whereas the sub-most effective and supra-most effective concentrations elicited hundreds of DEGs, suggesting that non-most effective concentrations trigger extensive transcriptional reprogramming of stress-response pathways. Layered Venn overlap analysis identified *PM1* as a potential candidate transcriptional marker of nicotinamide dose deviation, as its expression was maintained at levels similar to those of the salt–alkali-stressed CK specifically under 50 mg/L treatment. KEGG enrichment analysis of DEGs from the non-most effective comparisons highlighted the ko03110, ko00199, and ko04141 pathways, suggesting a putative, hypothesis-generating role of ER protein processing, detoxification, and oxidative stress-related transcriptional responses at non-most effective concentrations, which requires direct experimental validation. These findings provide a foundation for understanding the dose-dependent physiological and transcriptomic responses of soybean seedlings to nicotinamide under controlled saline–alkaline conditions. Further field studies are needed to evaluate the applicability of these findings to agricultural production.

## Figures and Tables

**Table 1:** RT-qPCR primer sequence.

Gene	F	R	Amplicon Length (bp)	Efficiency (%)	*R* ^2^
*PM1*	CACCACCGGGGAATATGGTC	CCCCCAGTCCCATACCCATA	207	98.6	0.997
*WRKY57*	GAAAGGGGAGTGCAAGGAGG	CAAACCAAACAAGGACATGCTCA	231	101.2	0.995
*XTH1*	TGAGTTCCGTGACCTTGACG	ATGTGAATGTGTGGCAGGGT	246	95.8	0.992
*LBD15*	CCTTTCCTCTTGCTGTGGTGT	ACTCACAGGGAGGTGAGCAA	295	97.3	0.996
*NAC1*	AGCCACCAAAGGGTCTCAAG	AGACCCAGTCATCTAGCCTCA	111	102.5	0.998
*GSTU43*	AGGGTATGGACAGGGAAGGG	TCCCACAAGTCTCAACCGTG	181	99.1	0.994
*HSP23*	ATGCCCGTCATCCTTTAGCC	GATCATCTTCGCGCTCCTCA	182	96.4	0.993
*HSP18*	AACGATAAGTGGCACCGTGT	TTGAGGCTTCTTCTCAGGCG	215	100.8	0.996
*CYP74A1*	ACCTTGTCGTTTCGAGCCAT	CCAGAAACAACCTGCACAGC	261	98.2	0.997
*CYP83D1*	TGACGGGAATTCTATCGCGG	GGAACTCGGGTCTGTTCCTG	248	97.7	0.995
*GAPDH*	GTCACAAGCCGTAGGAATCA	GCGGAAGTTGGTGAGAGATAG	107	99.5	0.999

**Table 2:** Effects of nicotinamide treatment on growth parameters of soybeans under saline–alkali stress.

Growth Indicators	Plant Height (cm)	Root Length (cm)	Stem and Leaf Fresh Weight (g)	Root Fresh Weight (g)	Stem and Leaf Dry Weight (g)	Root Dry Weight (g)
CK	16.4 ± 0.12 c	12.8 ± 0.10 c	2.033 ± 0.02 c	1.767 ± 0.03 c	0.467 ± 0.01 c	0.107 ± 0.01 c
SA1	19.3 ± 0.22 bc	18.3 ± 0.13 b	2.667 ± 0.04 bc	2.283 ± 0.02 b	0.533 ± 0.02 bc	0.137 ± 0.01 b
SA2	26.5 ± 0.24 a	19.3 ± 0.12 a	3.167 ± 0.06 a	2.507 ± 0.06 a	0.833 ± 0.03 a	0.167 ± 0.02 a
SA3	21.8 ± 0.18 b	17.3 ± 0.14 b	2.833 ± 0.05 b	2.167 ± 0.07 b	0.667 ± 0.04 b	0.133 ± 0.03 b

**Note: CK: saline–alkali-stressed control without nicotinamide treatment; SA1: 10 mg/L; SA2: 50 mg/L; SA3: 200 mg/L.** Data are presented as mean ± SD (*n* = 15 pots per treatment, with three seedlings per pot). Different letters within the same column indicate significant differences among treatments (one-way ANOVA followed by Tukey’s post hoc test, *p* < 0.05).

## Data Availability

The RNA-seq data generated in this study have been deposited in the Science Data Bank (https://www.scidb.cn) and are accessible via the DOI: 10.57760/sciencedb.42114. The data will be publicly released upon publication.

## Supplementary Materials

The following supporting information can be downloaded at: https://www.mdpi.com/article/10.3390/metabo16090649/s1, Table S1. Mapping statistics of RNA-seq samples. Table S2. DEGs between SA1 and CK. Table S3. DEGs between SA2 and CK. Table S4. DEGs between SA3 and CK. Table S5. DEGs between SA2 and SA1. Table S6. DEGs between SA3 and SA1. Table S7. DEGs between SA3 and SA2. Table S8. Pearson correlation matrix (FPKM). Table S9. Pearson correlation matrix (TPM). Figure S1. PCA plot of all samples (FPKM). Figure S2. Pearson correlation heatmap (FPKM). Figure S3. Expression distribution boxplots (FPKM). Figure S4. Density distribution plots (FPKM).

## Abbreviations

NAD	nicotinamide adenine dinucleotide
NADP	nicotinamide adenine dinucleotide phosphate
NBT	nitrogen blue tetrazolium
POD	Peroxidase
SOD	Superoxide Dismutase
CAT	Catalase
APX	Ascorbate Peroxidase
K^+^	potassium
Na^+^	sodium
O_2_^−^·	superoxide anion
KEGG	Kyoto Encyclopedia of Genes and Genomes
PCA	principal component analysis
GO	Gene Ontology
DEGs	differentially expressed genes
FC	Fold Change
ANOVA	One-way analysis of variance
SD	standard deviation
DCPIP	2,6-Dichlorophenolindophenol
FPKM	Fragments Per Kilobase of transcript per Million mapped reads
HSP	Heat Shock Protein
PM1	Plasma Membrane Protein 1 (a LEA protein family member)
PVP	Polyvinylpyrrolidone
UPR	Unfolded Protein Response
*XTH1*	Xyloglucan Endotransglucosylase/Hydrolase 1
*WRKY57*	WRKY Transcription Factor 57
*LBD15*	LATERAL ORGAN BOUNDARIES DOMAIN 15
*NAC1*	NAC Transcription Factor 1
*GSTU43*	Glutathione S-Transferase Tau Class 43
*HSP23*	Heat Shock Protein 23
*HSP18*	Heat Shock Protein 18
*CYP74A1*	Cytochrome P450 74A1
*CYP83D1*	Cytochrome P450 83D1
